# Early Renal Involvement in a Girl with Classic Fabry Disease

**DOI:** 10.1155/2017/9543079

**Published:** 2017-10-01

**Authors:** Fernando Perretta, Norberto Antongiovanni, Sebastián Jaurretche

**Affiliations:** ^1^Servicio de Terapia Intensiva del Hospital Dr. Enrique Erill de Escobar, Provincia de Buenos Aires, Argentina; ^2^GINEF Argentina (Grupo de Investigación Nefrológica en la Enfermedad de Fabry), Buenos Aires, Argentina; ^3^Centro de Infusión y Estudio de Enfermedades Lisosomales del Instituto de Nefrología Clínica Pergamino, Provincia de Buenos Aires, Argentina; ^4^Centro de Neurociencias Los Manantiales, Grupo Gamma Rosario, Provincia de Santa Fe, Argentina; ^5^Cátedra de Biofísica y Fisiología, Instituto Universitario Italiano de Rosario, Provincia de Santa Fe, Argentina

## Abstract

Fabry disease is an X-linked lysosomal storage disorder resulting from the deficiency or absence of the enzyme alpha galactosidase A; this defect leads to the systemic accumulation of globotriaosylceramide and its metabolites. Organic involvement in men is well known, but in women it is controversial, mainly due to the random X-chromosome inactivation in each of their cells (Lyon hypothesis). This would explain why women (heterozygotes) present a wide variability in the severity of their phenotype. The manifestations are multisystemic and begin in early childhood, reaching a severe compromise in adulthood. Typical acroparesthesia in hands and feet, gastrointestinal symptoms, angiokeratomas, dyshidrosis, hearing loss, arrhythmias, hypertrophic cardiomyopathy, cerebrovascular accidents, and renal failure can be observed. Nephropathy is one of the major complications of Fabry disease. Glomerular and vascular changes are present before progression to overt proteinuria and decreased glomerular filtration rate, even in pediatric patients. A case of incipient renal involvement in a girl with classic Fabry disease is reported.

## 1. Introduction

Fabry disease (FD) is caused by the lysosomal accumulation of complex glycosphingolipids, mainly globotriaosylceramide (Gb3) and its metabolites [1]. This deposit triggers physiopathogenic pathways in the vascular endothelium and cells of different tissues (cardiac, renal, and nervous among others) that lead to cell death, with progression to fibrosis and irreversible organic damage [2, 3]. The storage of Gb3 is due to the deficient or null activity of *α*-galactosidase A (*α*-galA, EC 3.2.1.22). The GLA gene, which encodes *α*-galA, is located on the X-chromosome (Xq22.1), whereby practically all men carrying a genetic mutation (hemizygous) develop the disease, while women (heterozygotes) exhibit a wide variability in the severity of their phenotype, mainly due to the random X-chromosomes inactivation in each of their cells (Lyon hypothesis) [4]. The symptoms intensity will depend mostly on the residual activity of the *α*-galA enzyme.

FD manifestations are multisystemic and begin in childhood, reaching severe impairment in the third or fourth decade of life. The main signs and symptoms of the disease are acroparesthesia in hands and feet, gastrointestinal disorders, angiokeratomas, dyshidrosis, intolerance to exercise and heat, hearing loss, arrhythmias, hypertrophic cardiomyopathy, cerebrovascular accidents, and renal failure [5, 6].

FD is panethnic and, given its low incidence, there is no accurate information regarding its prevalence, ranging from 1 : 40,000 men to 1 : 117,000 live births [7, 8]. Due to the great phenotypic and symptoms variability, it is difficult to perform a precise diagnosis, which is reached in adult ages when the organic involvement is already installed.

Thanks to systematic studies of FD detection in dialysis centers, it has been possible to advance in the determination of the prevalence in the dialysis population. It is 0.33% in men, showing that screening is a useful strategy in patients with chronic kidney damage [9].

Once the diagnosis has been made, it is possible to work on the family screening, which will allow the identification of affected relatives, thus detecting patients at an earlier age [10].

In the last years there has been progress in understanding the pathophysiology of tissue damage in FD, mainly in early organ involvement due to the accumulation of Gb3, where it was observed that asymptomatic pediatric patients already present tissue alterations [11]. This report describes incipient renal involvement in a pediatric patient with classic FD.

## 2. Case Presentation

A 9-year-old female patient was diagnosed with FD by family screening at 5 years of age, mutation c.1244T>C (p.L415P) in heterozygous status, alpha galactosidase in dried blood spot on filter paper of 1.5 umol/l/h (reference value ≥ 4.0).

**Figure 1:**
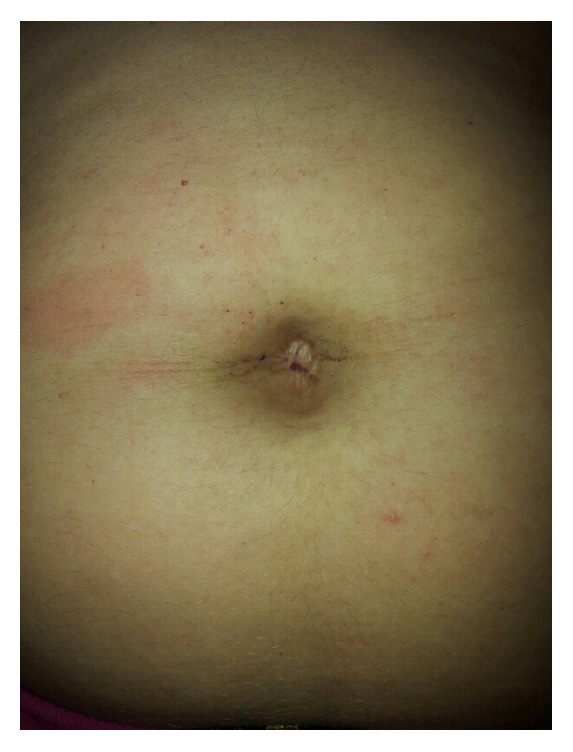
Periumbilical angiokeratomas.

It is a patient with normal physical and neurological development according to age. On examination, the following is found: preserved vital signs with normal blood pressure, weight 52 kg, height 151 cm, rare bronchospasms, mild acroparesthesias with good response to carbamazepine 200 mg/day, and few periumbilical angiokeratomas (Figure 1).

*Complementary Studies*. Laboratory findings are as follows: creatinine of 0.34 mg/dl (estimated glomerular filtration rate by Schwartz formula of 183 ml/min), albuminuria of 2.7 ug/min (reference value from 0 to 15), and proteinuria of 30 mg/24 hours (reference value < 150), with blood Lyso-Gb3 of 69.9 nmol/l (reference value < 1.2), normal electrocardiogram for their age, Doppler echocardiography with physiologic tricuspid and pulmonary regurgitation, normal brain magnetic resonance imaging, ophthalmological examination with slit lamp showing cornea verticillata in both eyes, and normal abdominal and renal ultrasound.

**Figure 2:**
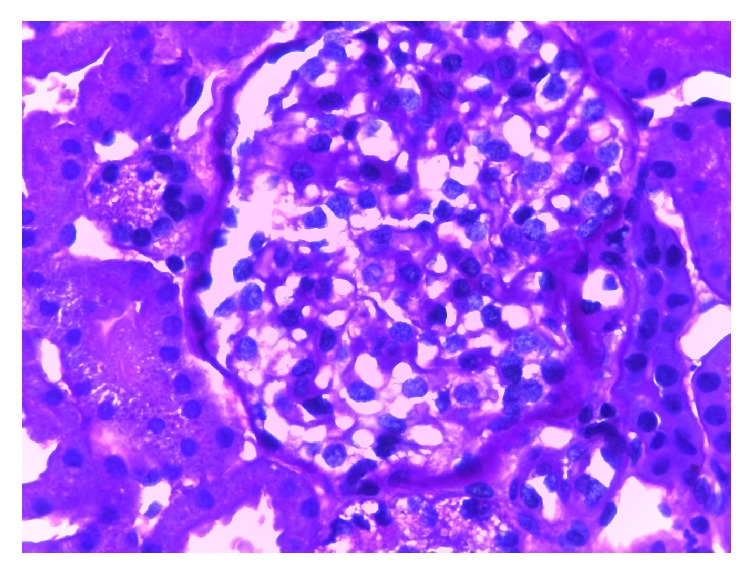
Light microscopy. PAS ×630.

**Figure 3:**
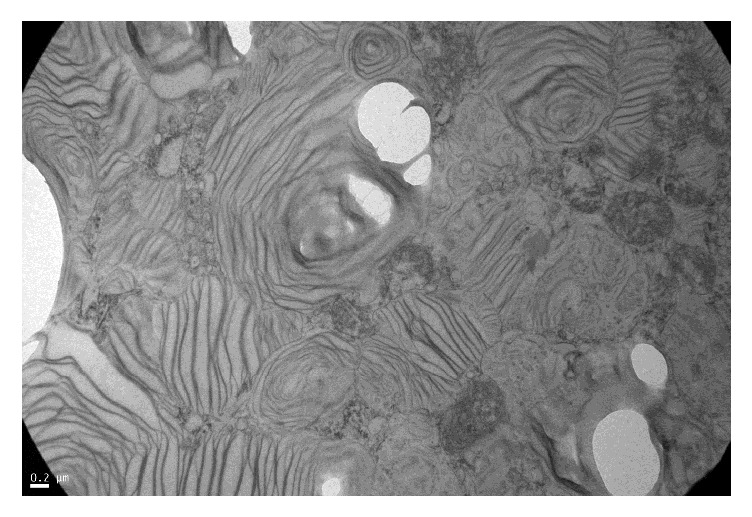
Electron microscopy ×25.000.

Although the patient did not present clinical data of nephropathy (albuminuria/proteinuria), due to the presence of glomerular hyperfiltration associated with peripheral neuropathy and an elevated blood Lyso-Gb3, a renal biopsy was performed. Light microscopy: after staining with hematoxylin and eosin, periodic acid-Schiff, Masson's trichrome, and silver-methenamine (Jones stain), glomeruli were observed with some degree of podocyte vacuolization that occupies on average 30% of the podocytes. Tubules: some distal tubules with clarification and microvacuolization of the cytoplasm. Interstitium and vessels were without alterations (Figure 2). Electron microscopy: glomeruli showed microvacuolization in one-micron sections (Fogo classification score 2), with mild clarification in the cytoplasm of proximal and distal tubules and Interstitium and vessels without alterations. Typical myeloid or zebra bodies were observed in the cytoplasm of several podocytes, confirming the diagnosis of FD (Figure 3). After a multidisciplinary evaluation, it was decided to start enzyme replacement therapy (ERT) with agalsidase beta at doses of 1 mg/kg body weight every 2 weeks by intravenous infusion.

## 3. Discussion

Gb3 deposits have been found in placental tissue, suggesting that the storage is already present at birth [12]. However, children are not symptomatic in the first years of life [13]. Patients generally present at an earlier age symptoms that manifest the progressive function loss of small nerve fibers of the peripheral somatic and autonomic nervous systems [13]. Early symptoms may include chronic neuropathic pain and/or acute attacks of pain (“Fabry crisis”); absence or decrease in sweating; tinnitus; intolerance to cold, heat, or exercise; gastrointestinal disorders (e.g., diarrhea, nausea, vomiting, postprandial bloating, and pain); and difficulty gaining weight. In addition, skin lesions (angiokeratomas), corneal and lenticular opacities, and the presence of mild proteinuria (in adolescent males) are among the first manifestations [14]. These symptoms generally cause morbidity despite the absence of major organ dysfunction, limiting the child's physical, school, and social performances. It has been reported that the symptoms can occur in early childhood, before the age of 5 years [15].

Screening is a valid tool to detect patients with FD, and performing a detailed pedigree, as in this case, can help to identify them at an earlier age before organic damage occurs [10].

Nephropathy is one of the major complications of FD [10]. Renal biopsies demonstrate Gb3 accumulation in tubular, glomerular, and endothelial cells, even in pediatric patients without albuminuria or decreased glomerular filtration rate [16, 17]. At present the only tool to detect early involvement is the kidney biopsy, but its routine indication is controversial because of the potential risks of the procedure. Percutaneous renal biopsy is safe in all ages when performed by experienced physicians; reduced estimated GFR and smaller center size are associated with an increased risk of major complications [18]. For this reason, the interest in the last years has focused on the study of noninvasive biomarkers able to detect early kidney damage. Lyso-Gb3 has been associated with neuropathic pain and with prealbuminuric histological changes, as in this case. Plasma measurement of Lyso-Gb3 is valuable for confirming the diagnosis of FD, particularly in heterozygous women, and its values correlate with disease severity [19]. A pilot study about the identification of urinary podocytes as a potential biomarker of glomerular damage, in primary and secondary glomerulopathies, was performed in Argentina [20]. Fabry disease is associated with increased podocyte loss; the direct associations found between podocyturia and proteinuria and the inverse association found between podocyturia and glomerular filtration rate in male patients indicate that there are important correlations between podocyturia and severity of Fabry nephropathy [21]. However, new biomarkers are needed to help in the stratification and quantification of renal damage in patients with FD to replace kidney biopsies.

In the present report, we describe renal histological involvement in a pediatric patient with classic FD and nonspecific signs and symptoms, prior to urinary protein loss (albuminuria/proteinuria). The kidney biopsy describes a score 2 of the Fogo et al. classification [16]; moderate podocyte vacuolization. This finding confirms what Tøndel et al. published in 2008; glomerular and vascular changes are present before progression to overt proteinuria and decreased glomerular filtration rate [11]. It has also been described that podocyte foot process effacement is an early marker of nephropathy in young classic Fabry patients without albuminuria. For this reason, renal biopsies may be essential in the early diagnosis of FD nephropathy and also in the evaluation of ERT response [17]. In this patient, we highlight the renal hyperfiltration values that can be interpreted as a glomerular compensation sign, despite the limitation that they were estimated by formula, but not measured.

In this case report, kidney biopsy provided useful information for the diagnosis of FD nephropathy, confirming the clinical suspicion. The lack of noninvasive biomarkers that correlate with the degree of tissue damage determines that, despite the controversies, a kidney biopsy is a necessary intervention in certain patients, even at pediatric ages.

Since 2001, FD has had a specific treatment with ERT, which has also demonstrated safety and efficacy [22]. It has been described that early histological renal lesions have a good response to ERT when it begins early. The clearance of Gb3 and the improvement of these lesions have been confirmed in serial kidney biopsies of pediatric patients treated with ERT and have a dose-dependent correlation [11, 17], and reaccumulation was observed after the decrease of the indicated dose [23].

To conclude, we emphasize the importance of suspecting FD and its exhaustive study. The kidney biopsy is an important tool in the assessment of renal involvement and can lead to the early initiation of ERT, which will change the natural history of this disease.
